# Effect of a Nanostructured Titanium Surface on Gingival Cell Adhesion, Viability and Properties against *P. gingivalis*

**DOI:** 10.3390/ma14247686

**Published:** 2021-12-13

**Authors:** Khaled Mukaddam, Monika Astasov-Frauenhoffer, Elizaveta Fasler-Kan, Laurent Marot, Marcin Kisiel, Ernst Meyer, Joachim Köser, Marcus Waser, Michael M. Bornstein, Sebastian Kühl

**Affiliations:** 1 Department of Oral Surgery, University Center for Dental Medicine Basel (UZB), University of Basel, Mattenstrasse 40, 4058 Basel, Switzerland; sebastian.kuehl@unibas.ch; 2 Department Research, University Center for Dental Medicine Basel (UZB), University of Basel, Mattenstrasse 40, 4058 Basel, Switzerland; m.astasov-frauenhoffer@unibas.ch; 3 Department of Biomedicine, University of Basel and University Hospital Basel, Hebelstrasse 20, 4031 Basel, Switzerland; e.fasler@unibas.ch; 4 Department of Pediatric Surgery, Children’s Hospital, Inselspital Bern, Department of Biomedical Research, University of Bern, Freiburgstrasse 15, 3010 Bern, Switzerland; 5 Department of Physics, University of Basel, Klingelbergstraße 82, 4056 Basel, Switzerland; laurent.marot@unibas.ch (L.M.); marcin.kisiel@unibas.ch (M.K.); ernst.meyer@unibas.ch (E.M.); 6 Institut für Chemie und Bioanalytik, Hochschule für Life Sciences, Hofackerstrasse 30, 4132 Muttenz, Switzerland; joachim.koeser@fhnw.ch (J.K.); marcus.waser@fhnw.ch (M.W.); 7 Department of Oral Health & Medicine, University Center for Dental Medicine Basel (UZB), University of Basel, Mattenstrasse 40, 4058 Basel, Switzerland; michael.bornstein@unibas.ch

**Keywords:** surface topography, nanocavity, biocompatibility, cell viability, implant fabrication

## Abstract

Objectives: The transgingival part of titanium implants is either machined or polished. Cell-surface interactions as a result of nano-modified surfaces could help gingival fibroblast adhesion and support antibacterial properties by means of the physico-mechanical aspects of the surfaces. The aim of the present study was to determine how a nanocavity titanium surface affects the viability and adhesion of human gingival fibroblasts (HGF-1). Additionally, its properties against *Porphyromonas gingivalis* were tested. Material and Methods: Two different specimens were evaluated: commercially available machined titanium discs (MD) and nanostructured discs (ND). To obtain ND, machined titanium discs with a diameter of 15 mm were etched with a 1:1 mixture of 98% H_2_SO_4_ and 30% H_2_O_2_ (piranha etching) for 5 h at room temperature. Surface topography characterization was performed via scanning electron microscopy (SEM) and atomic force microscopy (AFM). Samples were exposed to HGF-1 to assess the effect on cell viability and adhesion, which were compared between the two groups by means of MTT assay, immunofluorescence and flow cytometry. After incubation with *P. gingivalis*, antibacterial properties of MD and ND were determined by conventional culturing, live/dead staining and SEM. Results**:** The present study successfully created a nanostructured surface on commercially available machined titanium discs. The etching process created cavities with a 10–20 nm edge-to-edge diameter. MD and ND show similar adhesion forces equal to about 10–30 nN. The achieved nanostructuration reduced the cell alignment along machining structures and did not negatively affect the proliferation of gingival fibroblasts when compared to MD. No differences in the expression levels of both actin and vinculin proteins, after incubation on MD or ND, were observed. However, the novel ND surface failed to show antibacterial effects against *P. gingivalis*. Conclusion: Antibacterial effects against *P. gingivalis* cannot be achieved with nanocavities within a range of 10–20 nm and based on the piranha etching procedure. The proliferation of HGF-1 and the expression levels and localization of the structural proteins actin and vinculin were not influenced by the surface nanostructuration. Further studies on the strength of the gingival cell adhesion should be performed in the future. Clinical relevance: Since osseointegration is well investigated, mucointegration is an important part of future research and developments. Little is known about how nanostructures on the machined transgingival part of an implant could possibly influence the surrounding tissue. Targeting titanium surfaces with improved antimicrobial properties requires extensive preclinical basic research to gain clinical relevance.

## 1. Introduction

Peri-implantitis, which is characterized by an inflammatory reaction of the implant surrounding tissue, is caused by bacteria directly adhering onto the surface of implants [1,2]. It is quite a frequent finding with a weighted mean prevalence of 22% (CI: 14–30%) of dental implants [3]. Various therapeutic modalities have been advocated to treat peri-implantitis, but these are still a matter of controversy [4,5]. Since bacterial adhesion onto the implant surface is the initial step in the pathogenesis of peri-implantitis, it seems crucial to find strategies to minimize bacterial adhesion and colonization by modifying the surface topography of the implants. Nanostructured surfaces have been demonstrated to exhibit antibacterial effects by either mechanically destroying attached bacteria or reducing their adhesion [6,7,8,9]. This effect is mainly caused by specific nanostructures of spike-like nanopillars, which have the capacity of mechanically destroying the murein wall of bacteria [10,11,12,13]. Depending on the general shape in terms of length, width and distances between these pillars, different effects such as penetration, rupture of the membrane through stretching or buckling of the bacterial wall are discussed as the actual antibacterial effect [7]. Titania nanotubes with a diameter of 100 nm could successfully enhance gingival fibroblast proliferation and attachment while reducing the adhesion of *P. gingivalis* [14]. In this regard, there seem to be different targets in terms of how a nanostructure should be designed, and implants with such surfaces have not yet been introduced into the field.

Antimicrobial properties have been achieved using different methods, e.g., glancing angle sputter deposition, nanoimprint lithography, hydrothermal manipulation [15,16,17,18,19], or by etching procedures. The latter method is of special interest since such an etching step is compatible with the normal workflow during dental implant fabrication. Recently Wang et al., could show an increased soft-tissue adhesion after long-term acid etching of titanium [20]. Acid solutions, such as piranha solution, have been shown to result in a porous surface that could enhance bactericidal activity through its mechanical properties and chemical state [21]. Although several studies have shown antimicrobial properties in vitro, most tested the properties against *E. coli* [21,22,23,24]. However, in contrast to *P. gingivalis*, *E. Coli* is not associated with peri-implant diseases. Recently, a reduction in vital bacteria population for *P. gingivalis* of approximatively 70% was observed for a novel nanostructured titanium surface [6]. The surface was obtained by double-etching a commercially available implant surface (sand-blasted, large-grit and acid-etched (SLA) (Institute Straumann AG, Basel, Switzerland)) with 98% H_2_SO_4_/30% H_2_O_2_ mixtures. The nanostructure itself showed irregular nanocavities with an edge-to-edge diameter of approximately 10–20 nm. Interestingly, these structures revealed reduction of bacteria on nanostructured titanium in comparison to rough surfaced control materials (zirconia and titanium). Another group also reported an upregulated attachment and adhesion of fibroblasts on titania nanopores with micro- and nanotopography [25]. However, microstructured surfaces like SLA are prone to bacterial colonization which is why the transgingival region of dental implants is typically unstructured. This region would largely benefit from a bactericidal property since it is generally the entry region for bacterial infections in initial states of peri-implantitis. Therefore, it seems reasonable to investigate whether direct etching of a smooth and untreated titanium surface with 98% H_2_SO_4_/ 30% H_2_O_2_ mixtures (piranha etching), which was not sand-blasted and pre-etched, would result in a comparable bactericidal effect against *P. gingivalis* as a future target for nanostructuration of antimicrobial implant surfaces. The aim of the present study was to successfully create a similar nanostructured titanium surface with 10–20 nm cavities, to test the bactericidal properties of such an etched nanostructured titanium surface against *P. gingivalis* and to evaluate and compare the effect on cell proliferation and adhesion of gingival fibroblasts to machined titanium surfaces.

## 2. Materials & Methods

### 2.1. Preparation of Nanostructured Titanium Specimen

Two different types of custom-made 15 mm diameter titanium grade two discs were used in this in vitro study: nanostructured discs (ND) (test group) and machined discs (MD) (control group). ND were obtained by double-etching the surface of the discs with a 1:1 mixture of 98% H_2_SO_4_ and 30% H_2_O_2_ for 5 h (piranha etching) at room temperature (RT) [22,26,27] according to a previous study [6]. In contrast to the study mentioned, a smooth machined substrate was used instead of an SLA surface to obtain ND. MD have a polished surface and correspond to the machined part of tissue-level dental implants (Institut Straumann AG, Basel, Switzerland). Of each type, 115 samples were prepared for the subsequent experiments.

### 2.2. Characterization of the Surface Structure

The surface topography of both groups was analyzed by scanning electron microscopy (SEM) (*n* = 2) and the surface topography was additionally characterized by intermittent contact mode atomic force microscopy (AFM) (Flex-AFM, Nanosurf AG; Liestal, Switzerland) (*n* = 2) [28]. AFM measurements were performed in ambient conditions with super sharp SSS-NCL cantilever from Nanosensors. The frequency, the spring constant and tip radius of curvature were equal to: f = 170 kHz, k = 35 N/m and R = 2 nm, respectively. The direct measurements of the adhesion forces were performed in contact mode AFM [29]. Soft contact mode PPP-Cont cantilever from Nanosensors was used, with spring constant equal to k = 0.3 N/m. The adhesion measurements were performed at 50 sample spots characterized by different topography features. To statistically average adhesion forces between titanium surfaces and silicon, 50 force-distance curves were acquired on each MD and ND sample, characterized by different topography features. Ten force-distance curves were acquired on each sample spot.

### 2.3. Interaction between Oral Bacteria and Specimen (ND, MD)

*P. gingivalis* (ATCC 33277) was grown for 96 h in supplemented thioglycolate (anaerobic conditions) and harvested in a stationary growth phase. The bacteria were resuspended into simulated body fluid enriched with 0.2% glucose and exposed on ND and MD for 6 h, at 37 °C in static anaerobic conditions. Thereafter, the discs were gently dipped in 0.9% NaCl, and bacteria were either harvested and cultivated by conventional culturing on Columbia blood agar (BBL^TM^, BD Becton Dickinson; Allschwil, Switzerland) or fixed in 2% glutaraldehyde, dehydrated in stepwise increasing concentrations of ethanol, critical point dried and coated with 10 nm gold to be analyzed by SEM. Plates were incubated for 7 days at 37 °C in anaerobic conditions before colony forming units per disc were determined. The bactericidal properties of the materials were assessed by applying vitality staining (LIVE/DEAD BacLight Bacterial Viability Kit; ThermoFisher Scientific, Basel, Switzerland), and relative fluorescence was measured (Synergy HTX; BioTek Instruments GmbH, Luzern, Switzerland) at excitation wavelength 485 ± 20 nm (emission at 528 ± 20 nm for live cells and 635 ± 32 nm for dead cells). All experiments were performed three times with duplicate probes for conventional culturing and live/dead staining (*n* = 6) and once with duplicate probes for SEM (*n* = 2).

### 2.4. Interaction between Gingival Fibroblasts and Specimen (ND, MD)

#### 2.4.1. Cell Culture

Human gingival fibroblasts (HGF-1) were purchased from the ATCC (CRL-2014) and were grown in Dulbecco’s minimal essential medium (DMEM) supplemented with 10% FBS and 1% penicillin-streptomycin solution at 37 °C, 5% CO_2_ and 100% humidity according to the appropriate tissue culture collection protocol. For all experiments, the HGFs were used during passages 3–8. Fetal calf serum (FCS), DMEM and Trypsin EDTA solution were from Bioconcept (Allschwil, Switzerland). All other chemicals employed in this study were from Merck (Schaffhausen, Switzerland) and of the highest grade of purity. All cell culture experiments were performed in TPP (Trasalingen, Switzerland) plastic ware.

#### 2.4.2. MTT Assay

Thirty thousand HGF-1 cells were cultivated on discs in 24 well plates, 72 h later the MTT (0.1 mg/mL) was added and the cells were incubated for a further 4 h. The reaction was stopped by the addition of 125 µL DMSO. The supernatants were harvested and the optical density was measured at 590 nm, as described previously (Siegrist et al., 2017). Three independent experiments were performed in triplicate (*n* = 9).

#### 2.4.3. Immunofluorescence Microscopy

The expression of actin and vinculin in HGF-1 cells after incubation on discs was also confirmed by immunofluorescence experiments. Twenty thousand HGF cells were cultured on discs for 72 h, fixed with ice cold MetOH/acetone (1:1) for 15 min, washed with PBS and stained with rabbit anti-human actin or mouse anti-human vinculin antibodies. Alexa Fluor 647 goat anti-rabbit or Alexa Fluor 488 goat anti-mouse antibodies were used as secondary antibodies in these experiments. The nuclei were stained with DAPI. Three independent experiments were performed in triplicate. The images were collected and analyzed on Olympus BX-51 microscope with 20X objective using proprietary software. Three independent experiments were performed.

#### 2.4.4. Flow Cytometry

HGF-1 cells were incubated on discs for 72 h, then harvested and fixed with 2% PFA for 30 min at RT and permeabilized with 0.1% TX-100 for 5 min at RT. For staining anti-Actin (Biolegend, San Diego, CA, USA) and anti-Vinculin (Abcam, Cambridge, UK) antibodies conjugated to Alexa 488 were used. Unstained cells or cells stained with isotype-matched IgG antibody served as a negative control. All experiments were performed three times, using 24 MD and 24 ND in each experiment. Mean fluorescence intensities were quantitatively analyzed (10,000 events in each variant). The images were collected on Becton Dickinson FACS Calibur using Cell Quest Pro Software.

#### 2.4.5. Cell Morphology by Scanning Electron Microscopy (SEM)

4 × 10^4^ HGF-1 cells were seeded onto the specimen surface (ND and MD). After 24 h culture cells will have been washed twice with PBS and fixed with 2 % glutaraldehyde in PBS for 30 min. Glutaraldehyde was removed and free aldehyde groups quenched by the addition of 1 mL of 0.1 M glycine in PBS. Cells were washed twice with PBS and subsequently fixed with 2% osmium tetroxide in 0.1 M cacodylate buffer. Incubation for 30 min. Cells were washed twice with cacodylate buffer. Dehydration was performed with graded ethanol (twice with 50%, 70%, 90% and 100% ethanol for 2 min). Samples were critical point dried with CO_2_ (Critical Point Dryer, CPD 030, BAL-TEC, Pfäffikon, Switzerland), and sputtered (SCD 050, Sputter Coater, BAL-TEC AG, Lichtenstein) with approximately 50 nm Au-Pd to render the cells electroconductive. Cell morphology was visualized with SEM (ESEM XL30, Philips, Eindhoven, The Netherlands). SEM was performed once with duplicate probes (*n* = 2). 

#### 2.4.6. Statistical Analysis

Data were collected in an Excel sheet (Microsoft Corporation, Richmond, CA, USA) for descriptive analysis. Student’s *t*-test was applied (IBM^®^, SPSS^®^ Statistics software Version 26.0 (IBM Corp., Armonk, NY, USA)) to assess statistically significant differences between the colonization of *P. gingivalis* on ND and MD in the experiments conducted. The level of significance was set to *p* < 0.05.

## 3. Results

### 3.1. Characteristics/Morphology of the Nanostructured Titanium Sample Discs

**Figure 1:**
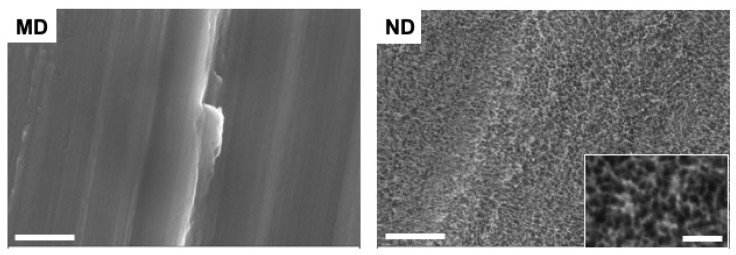
SEM images of MD and ND samples. ND shows an irregular nanostructure compared to MD. Nanocavities on ND have an edge-to-edge diameter of 10–20 nm. Scale bars: main images: 500 nm, inset: 100 nm.

The applied piranha etching process resulted in an irregular nanostructured surface for ND and created cavities with a 10–20 nm edge-to-edge diameter, while the machining lines remained, indicating a shallow etching process. MD shows a smooth surface, while acid etching of this surface resulted in an interconnected nanocavity structure (ND) (Figure 1).

**Figure 2:**
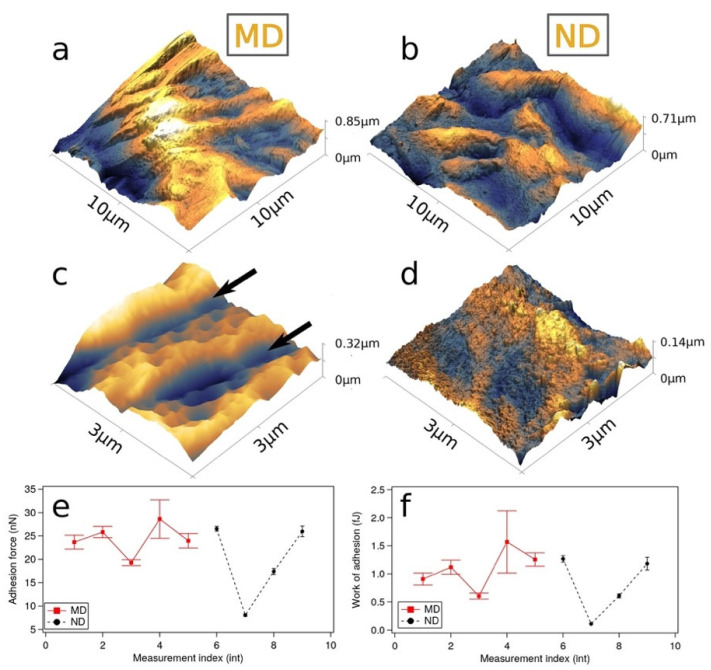
(**a**–**d**) AFM topography images of machined titanium samples (**a**,**c**) and H_2_SO_4_/H_2_O_2_ etched test object samples (**b**,**d**). 10 µm × 10 µm images showed surface spanning between 102 and 157 nm. Higher magnification images show trenches (marked by arrows) due to the machining process for machined (**c**) surface. The adhesion force and work of adhesion between AFM tip and titanium surfaces are shown in (**e**,**f**). Both surfaces show similar adhesion forces (see text).

Examples of the surface topography measured by means of intermittent contact AFM are shown in Figure 2a–d together with adhesion data (Figure 2e,f). The large scale 100 µm^2^ images (Figure 2a) reported surface rms roughness equal to 101 nm/157 nm and surface area 108 nm^2^/107 nm^2^ for MD/ND surfaces, respectively. Relevant surface topography parameters are summarized in Table 1.

The measured roughness values are consistent with the reported SEM data, and 100 nm nanostructures are visible for both surfaces as shown in Figure 2c,d. MD surface topography is characterized by the presence of trenches formed during the machining process (arrows in Figure 2c), while ND surface shows several few nm-sized features which is again consistent with SEM observations. The formation of several nm-sized cavities is clearly visible in 500 nm × 500 nm scale AFM images (not shown here). Both surfaces show similar adhesion forces equal to about 10–30 nN. Although adhesion is similar for both surfaces, the measurement for the etched surface showed a reduction of the statistical error, which we attribute to differences in surface morphology.

### 3.2. Interaction between Oral Bacteria and Specimen

**Figure 3:**
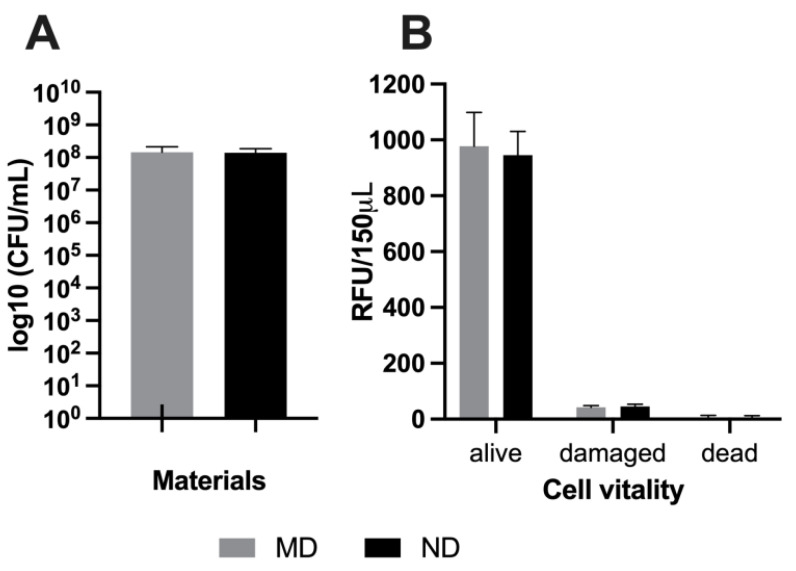
Detecting the antimicrobial properties of tested materials by determining the amount of *P. gingivalis* after 6 h of incubation on machined (MD) and nanosurfaced titanium (ND) by conventional culturing to measure the bacterial cells viable for cultivation (**A**) and vitality staining to assess the proportions of live and dead cells based on their membrane intactness (**B**). All experiments were performed three times with duplicate probes.

**Figure 4:**
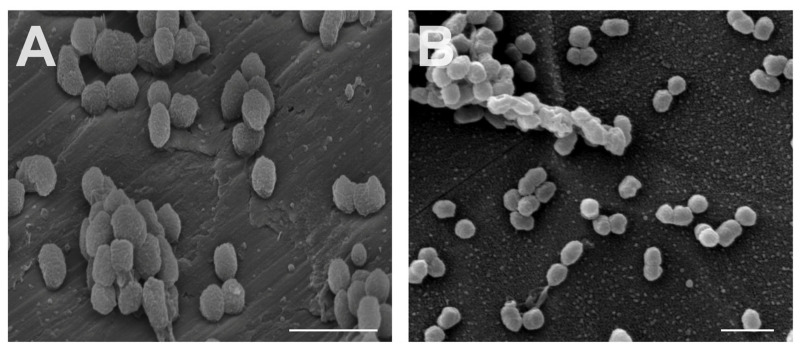
Scanning electron microscopy images of *P. gingivalis* cells on the two tested materials: MD (**A**) and ND (**B**). Bar indicates 1 µm.

The concentration of bacteria in the working solution was determined at the beginning (t = 0; 5.67 ± 3.40 × 10^9^ CFU/mL) and the end of the experiment (t = 6; 1.97 ± 0.61 × 10^9^ CFU/mL), exhibiting no statistically significant differences (*p* > 0.05) between experiments and also between the timepoints in bacterial counts. Additionally, no statistical difference was found between the ND and MD (*p* > 0.05; *n* = 9) after 6 h of bacterial adherence (Figure 3A) determined by conventional culturing. The bactericidal effect of the materials assessed by vitality staining (Figure 3B) revealed no statistical difference (*p* > 0.05). Additionally, no differences concerning the adhesion and structure of bacteria were detected between the two different surfaces on SEM images (Figure 4).

### 3.3. Interaction between Gingival Fibroblasts (HGF-1) and Specimen

#### MTT Assay

**Figure 5:**
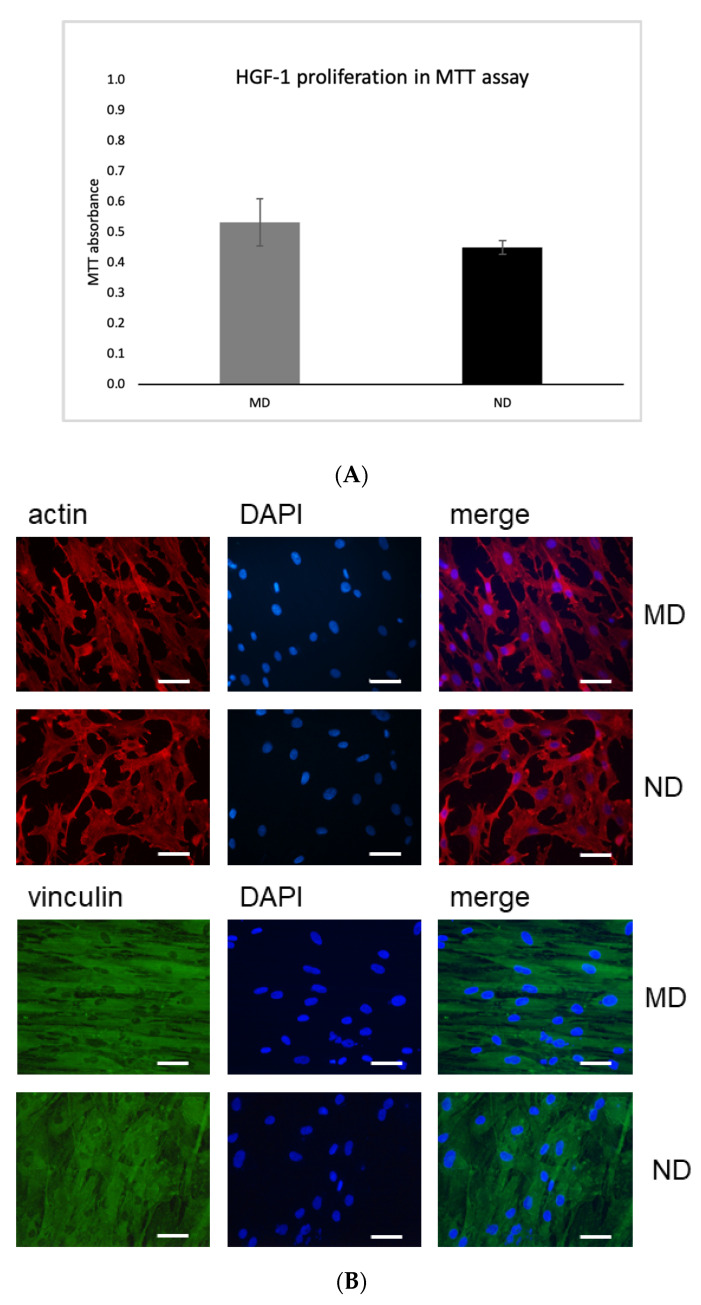

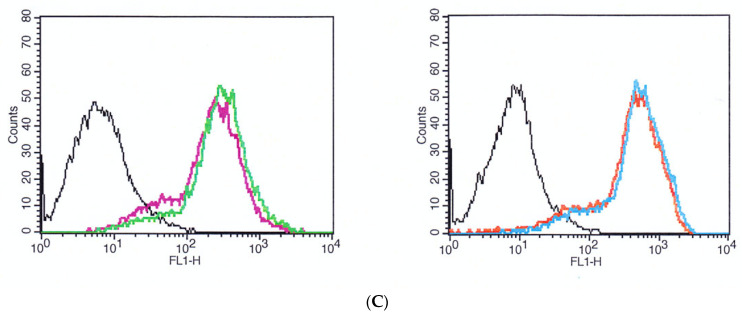
(**A**) Comparison of the MD and ND in MTT assay. Values represent mean ± SD. Three independent experiments were performed in triplicate, *p*-value 0.16. (**B**) Immunofluorescence staining of actin (upper panel) and vinculin (lower panel) on HGF-1 cells after 72 h incubation on MD and ND. Left: HGF-1 cells stained with anti-actin or anti-vinculin antibodies; center: DAPI staining; right: the merge between the actin/vinculin and DAPI images. Magnification 20×. Scale bar 50 µm. HGF-1 cells showed strong expression of actin (upper panel) and vinculin (lower panel). Cells show a flat morphology and appear adherent to the surface. On MD the HGF-1 cells had mainly spindle-shaped morphology, if they aligned along the grooves. Three independent experiments were performed in triplicate. (**C**) Flow cytometry analysis of HGF-1 cells cultivated for 72 h on MD or ND. Three independent experiments were performed, using 24 discs for each experiment. Left image: fluorescence intensities of HGF-1 cells incubated with anti-actin antibodies (pink histogram: HGF-1 cells incubated on MD; green: on ND). Right image: HGF-1 cells incubated with anti-vinculin antibodies (red histogram: on MD; blue: on ND). Black histograms: negative control (HGF-1 cells stained with isotype-matched IgG).

Incubation of HGF cells on MD and ND did not influence their proliferation capacity. Moreover, incubation on both discs showed very similar results, as absorbance peaks were almost identical between the two surfaces tested (Figure 5A).

### 3.4. Expression of Actin and Vinculin in HGF Cells Incubated on MD and ND Determined by Immunofluorescence Microscopy

Immunofluorescence studies allowed analysis of expression of actin (Figure 5B upper panel) and vinculin (Figure 5B lower panel) proteins in HGF-1 cells upon 72 h incubation on MD or ND. All discs revealed very similar results (Figure 5B). No differences in the expression patterns when the HGF cells were incubated on MD or ND were observed.

### 3.5. Expression of Actin and Vinculin in HGF-1 Cells upon Incubation on MD and ND by Flow Cytometry

Expression of actin and vinculin was also examined in HGF-1 cells after 72 h incubation on MD and ND by flow cytometry. As shown in Figure 5C, we did not observe any differences in the expression levels of both actin and vinculin after incubation on MD or ND.

Taken together the data indicate that MD and ND did not show substantial differences in the MTT assay as well as in immunofluorescence staining or flow cytometry.

### 3.6. Scanning Electron Microscope Images of HGF-1 Cells on Discs

**Figure 6:**
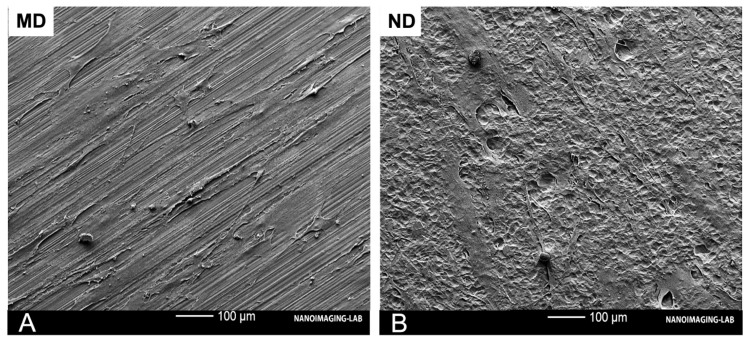
SEM images of HGF-1 after 24 h on the tested materials: MD (**A**) and ND (**B**). Abundant filodopia attachments were found on all samples. Portions of the HGF-1 cytoplasm exhibited a reticulated appearance.

Moreover, cell morphology was qualitatively assessed by SEM after 24 h of culture. SEM images of ND and MD showed that flattened and elongated HGF-1 cells adhered to the surface. Filodopia attachments were found on all samples. However, these attachments were more abundant on ND. MD shows spindle-shaped fibroblasts, whereas ND shows reticular-shaped HGFs (Figure 6).

## 4. Discussion

The present study demonstrates that direct etching of machined titanium with 98% H_2_SO_4_/30% H_2_O_2_ mixtures can successfully form nanocavities with an edge-to-edge diameter from 10 to 20 nm despite the absence of a sand-blasted, large-grit and acid-etched surface. The nanocavity surface did not have a negative impact or cytotoxic effect on the proliferation of fibroblast cells when compared to unstructured machined titanium surface. However, in contrast to promising results of a prior study with up to 70% reduction of vital bacteria, which were obtained by etching SLA surfaces additionally with the etching protocol employed in the present study [4], the nanocavity structure alone failed to show an antibacterial effect against *P. gingivalis.* One reason for the missing antimicrobial effect in the present study might be the varying macro- and microstructural topography of the surfaces. In contrast to the present surface, the nanostructure in the study of Astasov-Frauenhoffer et al., 2017 was applied on a macrorough SLA surface which showed additional macrostructuration of 20–40 µm caused by the sand-blasting procedure and the HCl/H_2_SO_4_ acid etching. The nanocavity structure in the present study was directly applied on a smooth machined surface and not on SLA surface, thus only providing the nanostructuration itself on a rather smooth underground. Obviously, the nanostructuration seems to have less impact on the bactericidal effect when compared to the combination of macro- and nanostructuration. The bactericidal characteristics of nanoporous surfaces are yet to be comprehensively studied. Here, the combination of macro- and nanoroughness results in different levels or vertical and horizontal distances between the single cavities leading to a more pronounced relief. Accordingly, due to these irregularities, stretching or buckling of the membrane causes higher tension and stress, facilitating rupture of the bacterial wall. Several groups have proven an antimicrobial effect on *E. coli*, *S. aureus*, *P. vulgaris* and other pathogens on nanostructured surfaces which had a similar design to our respective surfaces [14,21,30,31]. In the mentioned studies the initial adhesion of Gram-negative and Gram-positive bacteria was markedly inhibited in the first 6 h by the surface described above [10,27,30]. In contrast to these studies, the present study could not show any bactericidal effect. Aside from structural reasons this might also be explained by the different type of bacteria when comparing our results to those in the literature for similar nanostructures. One possible explanation could be the difference in size and diameter of *E. coli* (2 μm long, 0.25–1 μm diameter) compared to *P. gingivalis* (1.5 μm long, 1 μm diameter) which might require a different nanoporous design of the nano-micro-hierarchical surface to enhance the antimicrobial ability. Furthermore, other bacterial characteristics like structure and morphological properties, for example, membrane thickness or elasticity, could prevent a bactericidal effect or promote initial adhesion. With regard to the effect of antibacterial nanostructures, the murein thickness of bacteria plays a pivotal role. In 2012 Ivanova et al., showed that cicadae wings have bactericidal effects against *P. aeruginosa* with a kill rate of approximately 2.05 × 10^5^ CFU/(min × cm^2^) [19]. This surface typically consists of nanopillars or nanospikes of a diameter ranging between 50 and 250 nm, with different heights, spacing and densities. Compared to the nanocavity surface in the present study, the nanostructures are almost double in length, thus appearing to be more effective in rupturing the murein wall. Another group reported that nanostructures functionalized by glass substrates with 40 nm SiO_2_ nanoparticles decreased *Streptococcus mitis* and *Staphylococcus aureus* coverage of the sample [32]. While little is known about antimicrobial surfaces and their ideal structure for this purpose, the present study showed that it seems important to design a surface with irregularities in terms of irregular height levels, thereby increasing the mechanical stress on the bacteria murein wall.

Since most bacteria are surface-attached cells, understanding bacteria-surface interaction is essential for preventing peri-implant infections. Different approaches to altering titanium surfaces to inhibit or even kill bacteria have been reported. Antimicrobial active titanium surfaces have been generated by treatment of titanium surfaces with H_2_O_2,_ which was attributed to surface-bound superoxide radicals or a cooperativity between a photocatalytic active TiO_2_ surface and H_2_O_2_ [33,34]. In our experiments, we did not observe this effect, most likely because of an extensive washing step following H_2_SO_4_/H_2_O_2_ etching and the storage time between generation of the nanostructured surface and the antimicrobial assay. Furthermore, the surface composition of H_2_SO_4_/H_2_O_2-_etched titanium surfaces has been described as being nearly identical to titanium surfaces with a native oxide layer, which is why we do not expect an influence of different surface composition in our experiments [23,27]. Watson et al., demonstrated the bactericidal effect on *P. gingivalis*, caused by the micro- and nanosurface of gecko skin, which consists of spinules with a radius of curvature smaller than 20 nm and spacing in the sub-micron range [35]. Another group suggested that the hydrophilic surface texture of nanopillars on the aquatic larvae of the drone fly might inhibit biofilm formation and may even be bactericidal [36]. By using an alkaline hydrothermal process Diu et al., introduced a potent titanium nanostructured surface with two different phenotypes: brush and niche type nanowires with a diameter of approximately 100 nm. The reported surface seems to be more effective against motile bacteria like *E. coli* or *P. aeruginosa* than against non-motile bacteria (*S. aureus*) [17]. While nanopillars induce a deformation and penetrate Gram-positive and Gram-negative bacteria, Jenkins et al., could show that this antibacterial activity might be mediated by oxidative stress [13]. However, it remains unclear what effect these kinds of surfaces would have on oral pathogens, periodontal cells or osteoblast.

Furthermore, a possible reason for the different outcome in the present study and the study by Luo. et al., could be the choice of control group [27]. In contrast to our group in which the control was a machined surface, which is the gold standard surface for the transgingival part of an implant, their control group was microstructured. It is well known that a microstructured surface allows more bacterial adhesion than machined surfaces [37,38]. This could be the reason why our results for test and control group were quite similar.

Since soft tissue-implant interactions affect the susceptibility of bacterial infections, the effect of the nanostructuration on gingival fibroblasts was also tested. In this regard, the study showed that there was no statistically significant difference in proliferation/viability for HGF-1 cells incubated on the two differently structured titanium discs. Additionally, integration of the tissue surrounding a dental implant would be desirable. Vinculin and actin play an important role in cell-cell and cell-matrix adhesion. Both are an important component of focal adhesion, which is critical for tissue remodeling, cell migration and other homeostatic processes [39,40,41,42]. Our immunofluorescence experiments revealed that on MD the HGF-1 cells had a spindle-shaped morphology and were aligned along the grooves. In contrast, on piranha etched surfaces (ND) we observed mainly cells with reticular morphology. This could be explained by the partial removal of the machining structures on ND through the applied etching process (as shown in Figure 1 and Figure 6). These findings suggest that changes in cell alignment are regulated by nanogrooves and machining structures with the expected morphological differences [40]. Fibroblasts are able to respond to the micro- and nanotopography of the substratum surface [43,44]. This phenomenon was first described in 1911 as ‘contact guidance’, meaning the orientation, alterations in cell shape, polarity and alignment of the cell dependent on the micro- or nanostructure of different surfaces [45]. In vitro experiments have also shown that fibroblasts also conform to the topography of the material surface, probably leading to mechanical interlocking [46]. A recent study by Petrini et al., showed that gingival fibroblasts favored linear-like surfaces on a macrogeometry level. In our experiments, we observed that human gingival fibroblasts prefer the machining grooves and this might be a possible reason for the spindle-shaped appearance [43]. Consistent with our findings Gulati et al., could show cell alignment of HGF along the direction of the nanopores with strong anchoring evident by enhanced filopodia and stress fibers [47]. Recent findings suggest that nanostructured surfaces can enhance HGF viability, proliferation, adhesion to the biomaterial and extracellular matrix deposition. These functions could be enhanced and triggered simply through the physico-mechanical effect of different micro- and nanoscale levels [48]. Thus, the pore diameter, geometry and organization of the nanostructured surface influence the cellular behavior of human gingival fibroblast and osteoblast [49]. Miao et al., compared the cellular response of an implant surface at the macro-, micro- and nanoscale level involving osteoblasts, epithelial cells, macrophages and fibroblasts [50]. Their results showed that nanostructures were beneficial for almost all cell types. However, fibroblasts did not show advantages when compared between micro- and nanoscale. These findings are in line with our results despite the fact that there was no dominant difference in this study. One reason for this might be the shallow etching profile which did not remove the machined lines of the untreated sample. The literature provides initial evidence that cells extend more filodopia on nanoporous titanium surfaces, which could be a promoting factor of gingival adhesion [14,49].

With regard to the potential positive effects that nanostructures might have on soft tissue integration with additional antimicrobial properties, further research is required. Although the present study failed to present such a surface, the results might contribute to better understanding of morphologies with antimicrobial properties.

## 5. Conclusions

Nanocavities of 10–20 nm on homogeneous titanium discs are insufficient and do not hinder *P. gingivalis* adhesion while supporting the adhesion of gingival fibroblasts. This needs to be considered in the development of alternative nanostructured surfaces targeting oral pathogens. Further studies on the strength of the gingival cell adhesion should be performed in the future. However, various nanostructuration processes combined with macro- and microalterations are promising techniques for achieving soft tissue integration of the peri-implant mucosa.

## Figures and Tables

**Table 1:** Topography parameters for MD and ND samples obtained by AFM. Several parameters were extracted based on image analysis, namely: surface area, surface roughness (Sq, Sa), skewness of the height distribution (Ssk) as well as max. peak height (Sp), max. pit depth (Sp) and max. height (Sz).

	MD Sample		ND Sample	
Projected area	100 nm^2^	9 nm^2^	100 nm^2^	9 nm^2^
Surface area	108.3 nm^2^	9.58 nm^2^	107.2 nm^2^	9.67 nm^2^
rms roughness Sq	101.7 nm	17.27 nm	157.4 nm	49.77 nm
Mean roughness Sa	82.6 nm	13.30 nm	130 nm	39.68 nm
Skew Ssk	0.084	0.1208	−0.2236	0.08191
Max. peak height Sp	405.5 nm	68.2 nm	371.5 nm	219.1 nm
Max. pit depth Sp	304.9 nm	70.8 nm	478.1 nm	134.8 nm
Max height Sz	710.5 nm	139 nm	849.6 nm	353.9 nm
